# Databases in veterinary medicine – validation, harmonisation and application: introduction

**DOI:** 10.1186/1751-0147-53-S1-S1

**Published:** 2011-06-20

**Authors:** Hans Houe, Agneta Egenvall, Anna-Maija Virtala, Thorstein Olafsson, Olav Østerås

**Affiliations:** 1 Faculty of Life Sciences, University of Copenhagen, Denmark; 2 Swedish University of Agricultural Science, Sweden; 3 University of Helsinki, Helsinki, Finland; 4 Icelandic Food and Veterinary Authority, Iceland; 5 The Norwegian School of Veterinary Science, Oslo, Norway

## 

In order to use all the stored information on animal diseases in an optimal manner it is crucial that the purpose, circumstances and constrains for the data are known for the user. The objective with the NKVet symposium and ensuing special issue of Acta Vet Scand is to provide an overview of the most important databases on animal diseases in the Nordic countries. The possibility of using the databases for different purposes such as animal welfare, food safety and animal health economics will be in focus with the special question if we, in the future, can make databases more transparent by establishing database protocols to share among users of the databases. Finally, the possibilities of harmonisation of databases will be addressed.

Large centralised data bases with information on animal diseases are becoming more and more common. The information can either be directly related to recording of the disease or pathogen, or it can be of indirect importance such as for example animal movements being a risk factor for disease transmission. The most detailed databases have been established for the production animals (cattle, pigs and poultry), but recently there has been increasing interest also for other species such as small animals and horses. Many databases have initially been established for different purposes such as, for example, calculation of breeding values in cattle or recording of specific pathogens to demonstrate disease freedom. The databases have been further elaborated over several years according to the specific circumstances, constraints and needs for the specific species. Therefore, the type of data on diseases varies from clinical observations, treatments pathological changes observed at meat inspection or laboratory diagnostic information on pathogens or their antibodies. Further, there is huge variation in events that trigger the collection of data being either a disease that needs treatment or a random sampling of animals. Obviously, this means that all the data on diseases have different validity in giving information on disease severity in terms of production loss or welfare or in terms of food safety.

## Competing interests

The authors declare that they have no competing interests.

